# Perilipin 5 Reduces Oxidative Damage Associated With Lipotoxicity by Activating the PI3K/ERK-Mediated Nrf2-ARE Signaling Pathway in INS-1 Pancreatic β-Cells

**DOI:** 10.3389/fendo.2020.00166

**Published:** 2020-03-31

**Authors:** Yunxia Zhu, Chenxi Ren, Mingliang Zhang, Yuan Zhong

**Affiliations:** ^1^Department of Geriatrics, Shanghai Jiao Tong University Affiliated Sixth People's Hospital, Shanghai, China; ^2^Department of Endocrinology and Metabolism, Shanghai Jiao Tong University Affiliated Sixth People's Hospital, Shanghai, China

**Keywords:** perilipin5, oxidative stress, pancreatic β-cells, free fatty acid, Nrf-2, MAPK

## Abstract

Oxidative stress induced by free fatty acid overload in pancreatic β-cells is a potential contributory factor to dysfunction of insulin secretion and apoptotic cell death. Perilipin 5 (Plin5) has been reported to ameliorate oxidative stress-mediated damage in non-insulin-secreting tissues. We tested the hypothesis that Plin5 plays a similar role in pancreatic β-cells, which are extremely sensitive to oxidative stress. Here, our *in vitro* data showed that Plin5-mediated alleviation of palmitate-triggered apoptosis involves the mitochondrial pathway. And the protective role of Plin5 on β-cells was partially dependent on its modulation in oxidative stress. Upregulation of *Plin5* in INS-1 cells decreased reactive oxygen species production, enhanced cellular glutathione levels, and induced expression of antioxidant enzymes glutamate-cysteine ligase catalytic subunit and heme oxygenase-1. However, knocking out of *Plin5* abolished all of these beneficial effects. Furthermore, by using the O^2−^ scavenger MnTMPyP, we verified that altering *Plin5* expression impacted lipotoxic cell death partially via modulating oxidative stress. Mechanistic experiments revealed that Plin5 induced Nrf2-ARE system, a master regulator in the cellular adaptive response to oxidative stress, by activating PI3K/Akt and ERK signal pathways, contributing to the increase of antioxidant defense and consequently improving β-cell function and survival in the presence of lipotoxic oxidative stress. Overall, our findings indicate that Plin5 abrogates oxidative damage in INS-1 β-cells during lipotoxic stress partially through the enhancement of antioxidant defense involving the PI3K/Akt and ERK mediated Nrf2-ARE system.

## Introduction

Loss of pancreatic β-cell function caused by reduced secretory capacity and enhanced apoptosis is central to the development and progression of type 2 diabetes (T2D) (1). Why β-cells progressively fail in T2D is not thoroughly understood. Evidence has indicated that chronically elevated circulating levels of saturated free fatty acids (FFAs), of which palmitate is the physiologically most abundant long-chain saturated FFA, are associated with T2D risk (2). Excess fatty acids accumulation in multiple non-adipocyte cell types, such as pancreatic β-cells, may result in metabolically relevant cellular dysfunction and injury, which is now termed “lipotoxicity” (3). However, despite the advances have been made, the molecular mechanisms involved in this phenomenon are still not fully clear.

Oxidative stress, a persistent deleterious imbalance between the production and the removal of free radicals including reactive oxygen species (ROS), has been considered to be an important factor in β-cell demise associated with lipotoxicity (4). On the one hand, pancreatic β-cells are known as hypersensitive targets for oxidative stress-mediated tissue damage because of the relatively low expression of antioxidant enzymes and non-enzymatic antioxidant defense (5, 6). On the other hand, generation of ROS in mitochondria, usually accompanied by FFA overload, leads to augment in oxidative stress in pancreatic β-cells (7). In support of this concept, experimental evidences have suggested that ROS inhibition by antioxidants ameliorates palmitate-induced cell death in INS-1 β-cells (8, 9). While fatty acid catabolism via mitochondrial β-oxidation is an important fuel source for pancreatic β-cells, excessive fatty acid oxidation is usually closely associated with enhanced ROS formation that may lead to lipotoxicity (10, 11). To reduce the lipotoxicity generated by fatty acid β-oxidation, fatty acids consumed by pancreatic β-cells can be converted into triglycerides and stored in lipid droplets (LDs) which are considered to be less toxic than FFA (12). Therefore, maintaining intracellular lipid metabolism homeostasis, a complex process which was linked to LDs dynamics, is a promising strategy to impede damages in pancreatic β-cells mediated by oxidative stress.

LDs, highly conserved across species and stored in virtually every cell type in humans, are now recognized as highly active organelles comprising of a hydrophobic core of neutral lipids surrounded by a phospholipid monolayer which is embedded with hundreds of LD-associated proteins (13). In non-adipose tissues, LD-associated proteins play a critical role in maintaining lipid metabolism homeostasis by regulating LD lipolysis (14). They not only supply fuel during periods of energy needs but also act as a lipolytic barrier to avoid cellular lipotoxicity (14). Therefore, dysregulated lipid droplet metabolism is tightly linked to numerous metabolic diseases including T2D (15). The metabolic regulation of LDs is mediated by LD-associated proteins that concentrate at the LD surface. The Perilipin family (Plins) is a representative group of LD-associated proteins with five members (Plin1–5). Among these, Perilipin 5 (Plin5) is mainly expressed in tissues with very active lipid catabolism, including heart, brown adipose tissue, skeletal muscle, and liver and promotes LD accumulation but inhibits lipolysis by interacting with lipases such as ATGL, Abhd5 and CGI-58 (16–20). By modulating lipid homeostasis, Plin5 plays a role in ameliorating oxidative stress-induced damage in heart, skeletal muscle, hepatic stellate cells and aortic valve tissues (21–25). However, the regulation of Plin5 in FFA-induced oxidative stress and oxidant damage in pancreatic β-cells, to our knowledge, is not established until now. In previous work including ours', Plin5 was found to play the pivotal role in β-cell survival and function (26, 27). Here, we hypothesize that Plin5 may exert protective effects on pancreatic β-cells under lipotoxic conditions by modulating the cellular oxidative stress levels. To test this hypothesis, we treated INS-1 β-cells with palmitate to mimic physiological oxidative stress. In addition, we aimed to demonstrate the potential molecular mechanism and signaling pathway involved in the process.

## Materials and Methods

### Cell Culture and Treatments

The rat insulinoma INS-1 cell line kindly provided by Dr. Christopher B. Newgard (Duke University Medical Center, USA) was routinely passaged and cultured as described previously (28). INS-1 β-cells between passages 20–30 were used. Each cell experiment was performed in triplicates with different passage of cells. For FFA exposure, palmitate (Sigma-Aldrich, USA) was dissolved in 99% ethanol to a concentration of 100 mM, and then mixed with 10% BSA in serum-free DMEM to make a 5 mM palmitate stock solution and used at a final concentration of 0.5 mM. MnTMPyP (MerckSharp & Dohme, USA) was diluted in water. It was added together with palmitate and maintained in the medium for 24 h after adenovirus transduction. To verify whether Plin5 was associated with PI3K/Akt or ERK pathways, LY294002 (10 μM, PI3K/Akt inhibitor, Millipore, USA) or U0126 (20 μM, ERK inhibitor, Calbiochem-Novabiochem, USA) was used 1 h before palmitate treatment and INS-1 cells treated with DMSO was used as the control.

### Adenovirus Transduction and siRNA Interference

Adenoviruses carrying rat Plin5 (Ad-Plin5) or GFP (Ad-GFP) and adenoviral Plin5 shRNA (Ad-shPlin5) were custom made at Shanghai Asia Vector Biotechnologies (Shanghai, China). Briefly, for construction of Ad-Plin5, cDNA fragment of PLIN5 was chemical synthesized and then ligated into the pTRACK-CMV vector. For Ad-shPlin5, fragment of Plin5 shRNA (5′-GGCAAGCACACAATGATGC-3′) was chemical synthesized and then ligated into the pTRACK-WT vector. The vectors were linearized by Pme I digestion and transfected into 293A cells for virus production and amplification. Adenovirus overexpressing GFP (Ad-GFP) was the control. The viruses were purified by CsCl density gradient centrifugation and the adenoviral titers were measured by using QuickTiter Adenovirus Titer Immunoassay Kit (Cell Biolabs, USA) according to the manufacturer's protocol. Generally, we used 20–40 multiplicity of infection (MOI) for overexpression and 50–100 MOI for shRNA knockdown experiments. For RNAi, the Nrf2-specific siRNA and scramble siRNA were designed and synthesized by Shanghai Genechem (Shanghai, China). The sequences of si-Nrf2 were 5′-CCGGAGAAUUCCUCCCAAUTT-3′ (sense) and 5′-AUUGGGAGGAAUUCUCCGGTT-3′ (antisense). SiRNA interference was performed with Lipofectamine 2000 according to the manufacturer's protocol. The efficiency of Ad-Plin5, Ad-shPlin5 and Nrf2-siRNA were confirmed by western blotting analysis.

### Assessment of β-Cell Apoptosis

Apoptotic cells were counted using an Annexin-FITC Apoptosis Detection Kit (Beyotime, China) according to the manufacturer's protocols and then were analyzed immediately by FACS Calibur flow cytometry. Apoptosis was confirmed by cytochrome *c* release and Caspase-3 cleavage by western blot.

### Dichlorofluorescin Diacetate (DCFH-DA) Assay

Intracellular oxidants were quantified by the DCFH-DA assay. Briefly, cells plated in 6-well plates were incubated with different adenoviruses and palmitate for indicated time. After that, 10 μM DCFH was added to the wells for 20 min at 37°C and then, the unabsorbed probe was removed. After being oxidized by intracellular oxidants, DCFH will become DCF and emit fluorescence. DCF fluorescence was quantified using a flow cytometry with an excitation wavelength of 480 nm and an emission wavelength of 525 nm (BD Biosciences, USA).

### Insulin Secretion Assay

INS-1 cells were transfected with adenoviruses and (or) siRNA and then incubated in RPMI 1640 medium containing glucose or palmitate at indicated concentrations. Glucose stimulated insulin secretion (GSIS) was performed as we previously described, with minor modifications (28). Briefly, after treatments, INS-1 cells were starved for 2 h in glucose-free Krebs-Ringer bicarbonate buffer (KRB, pH 7.4) and then incubated in KRB buffer containing 2.5 or 25 mM glucose for 1 h. Insulin secreted in the medium and the insulin content in the cell were assayed by a rat insulin ELISA kit according to the manufacturer's instructions (X-Y Biotechnology, China).

### Western Blot

After different treatments, INS-1 cells were quickly harvested, rinsed thoroughly with PBS for two times, and then lysed in ice-cold RIPA lysis buffer containing protease and phosphatase inhibitors (JRDUN, China). The lysates were collected by centrifugation (12,000 rpm, 10 min). The supernatants were assembled, and protein concentrations were measured using a BCA Protein Assay Kit (Thermo Scientific, USA). For the detection of cytochrome *c* release, a NE-PER kit (Thermo Scientific, USA) was used to separate the mitochondrial and cytosolic fractions. For the measurement of nuclear Nrf2 protein content, after collecting the supernatant fractions (cytosolic cell extracts). the resulting pellets containing crude nuclei were suspended in 50 μL extraction buffer containing 20 mM HEPES (pH 7.9), 400 mM NaCl, 1 mM EDTA, 10 mM dithiothreitol, and 1 mM PIC, and then kept on ice for 30 min. Samples were centrifuged at 14,000 rpm 4°C for 10 min to obtain supernatants containing nuclear cell extracts. For western blots, equal amounts of proteins (25 μg) were separated by 10 or 15% gel electrophoresis and were electrophoretically transferred to polyvinylidene difluoride (PVDF) Immobilon membranes. Then the membranes were blocked with 5% skim milk and incubated with the corresponding primary antibody followed by incubation with secondary antibody. The primary antibodies were anti-cytochrome *c* (1:1000), cleaved Caspase 3 (1:500), glutamate-cysteine ligase catalytic subunit (GCLC, 1:1000) and heme oxygenase-1 (HO-1, 1:2000) from Abcam (USA), PLIN5 (1:1000) from Progen Biotechnik (Germany), Tom20 (1:500) from Santa Cruz (USA) and Histone H3 (1:1000), p-Akt (1:2000), Akt (1:1000), p-p38 (1:1000), p38 (1:1000), p-ERK1/2 (1:1000), ERK1/2 (1:1000), p-JNK (1:1000), JNK (1:1000), Nrf2 (1:1000), GAPDH (1:1000) from CST (USA). The secondary antibodies were horseradish peroxidase-labeled goat anti-rabbit (1:1000), goat anti-rat (1:1000) or donkey anti-goat (1:1000) (Beyotime, China). Protein signal were detected using an electrochemiluminescence detection kit (Millipore, USA) and quantified using Image J software (NIH, USA). Equal loading of proteins was verified by GAPDH or Tom20.

### Measurement of Glutathione (GSH) Content

Cell pellets were lysed by a homogenizer in cold PBS. Following centrifugation at 4,000 rpm for 15 min at 4°C, the supernatant was harvested for the measurement of cellular GSH using a commercial kit according to the manufacturer's instructions (Nanjing Jiancheng Bioengineering Institute, China). Briefly, GSH was spectrophotometrically measured (at 420 nm) and the results were expressed in mg/g prot.

### Plasmids and Promoter Reporter Assay

INS-1 cells were co-transfected with the ARE-luciferase report construct (750 ng, Biovector, China) and the Renilla luciferase reporter vector plasmid pRL-TK (50 ng, Promega, USA) using Lipofectamine 2000 (Invitrogen, USA) for 6 h. After that, the cells were transfected with different adenoviruses and (or) Nrf2-siRNA for 18 h, after which 0.5 mM palmitate was added for another 24 h. Subsequently, the cells were washed and lysed according to the manufacturer's instructions. The luciferase activity of cell lysates was measured using a GloMax®-Multi+ Detection System (Promega, USA) and was expressed as relative luciferase/Renilla activity.

### Immunofluorescence

Cells were seeded on polylysine coated cover slips and allowed to attach overnight. Following treatments, cells were washed in PBS, fixed with 4% paraformaldehyde, permeabilized with 0.5% Triton X-100 and incubated overnight with primary antibody against Nrf2 (Abcam, USA). After washing three times with PBS, cells were incubated with second antibody conjugated with Alexa fluor 488 (Biosea, China) for 1 h at room temperature, nuclei stained with 4,4-diamidino-2-phenylindole (DAPI, 1:500). The slides were then observed by a fluorescence microscope (Leica, DM6000B).

### Statistical Analysis

Data are presented as means ± SEMs and represent the results of at least 3 independent experimental trails. The significance of differences was determined by either unpaired Student's *t*-test or One-Way ANOVA where applicable using GraphPad Prism 5.0 (GraphPad Software, USA). The value of statistical significance was set at *p* < 0.05.

## Results

### Plin5 Protects Against Lipotoxicity in INS-1 β-Cells and Involves the Mitochondrial Pathway

**Figure 1:**
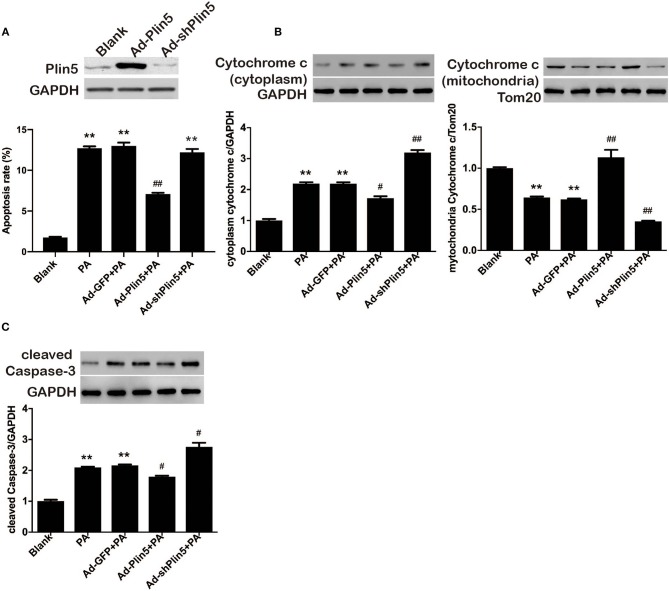
Plin5 protects against lipotoxicity in pancreatic β-cells involves the mitochondrial pathway. **(A)** Up, Plin5 expression in INS-1 cells was overexpressed or suppressed using Ad-Plin5 or Ad-shPlin5 for 24 and 36 h, respectively. Protein expressions were determined by Western blots. GAPDH served as an invariant control for equal loading. Down, the apoptosis rate was determined using Annexin V assay in INS-1 cells without any treatment (Blank) or transfected with Ad-GFP (24 h), Ad-Plin5 (24 h), or Ad-shPlin5 (36 h) and then exposed to 0.5 mM palmitate (PA) for another 24 h. Data are expressed as the mean ± SEM (*n* = 4); **P < 0.01 against untreated cells (Blank); ^##^*P* < 0.01 against PA group. **(B)** Cytoplasmic (up) and mitochondrial (down) cytochrome *c* protein expression in INS-1 cells without any treatment (Blank) or transfected with Ad-GFP (24 h), Ad-Plin5 (24 h), or Ad-shPlin5 (36 h) and then exposed to 0.5 mM palmitate (PA) for another 24 h. Protein expressions were determined by Western blots. Data are presented as the mean ± SEM (*n* = 4) and expressed as the fold change taking Blank group as 1. GAPDH and Tom20 served as loading control for the cytoplasmic and mitochondrial fractions respectively. **P < 0.01 against Blank; ^*##*^*P* < 0.01, ^#^*P* < 0.05 against PA group. **(C)** Cleaved Caspase-3 protein expression in INS-1 cells treated as described in A. Protein expressions were determined by Western blots. Data are presented as the mean ± SEM (*n* = 4) and expressed as the fold change taking Blank group as 1. GAPDH served as loading control. **P < 0.01 against Blank; ^#^*P* < 0.05 against PA group.

Plin5 serves as an LD protein in human islets, mouse islets and pancreatic β-cell lines (MIN6 and INS-1) (26, 27), however its concrete role in β-cell's function and survival is rarely defined. We previously reported that upregulation of Plin5 decreased cell apoptosis, and hence protected β-cells from palmitate-induced lipotoxicity by alleviating endoplasmic reticulum stress in pancreatic β-cells (26). Nevertheless, endoplasmic reticulum stress cannot be used to entirely explain the differences in cell apoptosis observed between Plin5-overexpression INS-1 cells and the control cells because its improvement only blunts the lipotoxic effects, not ablates them. Palmitate-triggered apoptosis typically involves the mitochondrial pathway which induces the release of cytochrome *c* from mitochondria into the cytosol (29). Cytosolic cytochrome *c* can lead to the activation of caspases in the apoptosome and ultimately to the activation of Caspase 3, which subsequently results in cell apoptosis. Therefore, we investigated whether the protection effect of Plin5 in INS-1 cells undergoing palmitate-induced apoptosis is due to the blocking of the intrinsic (mitochondria) pathway by evaluating the cytochrome *c* release and activation of Caspase 3, as Plin5 which also exists in mitochondria in addition to localizing to LD, cytosol, and endoplasmic reticulum may involve in the regulation of mitochondria biogenesis and function (30–32). To this end, we used loss- and gain- of-function approaches. Plin5 expression and its overexpression or deletion by adenovirus transduction were confirmed by Western blot in INS-1 β-cells (Figure 1A, the upper side). In the present study, overexpression of Plin5 alleviated palmitate-induced apoptosis, which is consistent with our previous study (Figure 1A) (26). Interestingly, deletion of Plin5 in INS-1 cells didn't augment lipotoxicity, which may be due to the relatively low expression of Plin5 in pancreatic β-cells compared with other members of Plins family (33). Furthermore, prolonged palmitate treatment resulted in a substantial increase in cytochrome *c* release from mitochondria and activation of Caspase 3 (Figures 1B,C), aligning with previous reports (34). Strikingly, palmitate-mediated mitochondrial dysfunction was decreased dramatically in INS-1 cells with *Plin5* overexpression yet potentiated in INS-1 cells with *Plin5* deficiency (Figures 1B,C). These results suggest that Plin5 modulates apoptosis associated with mitochondria pathway.

### Plin5 Modulates Oxidative Stress in INS-1 β-Cells

**Figure 2:**
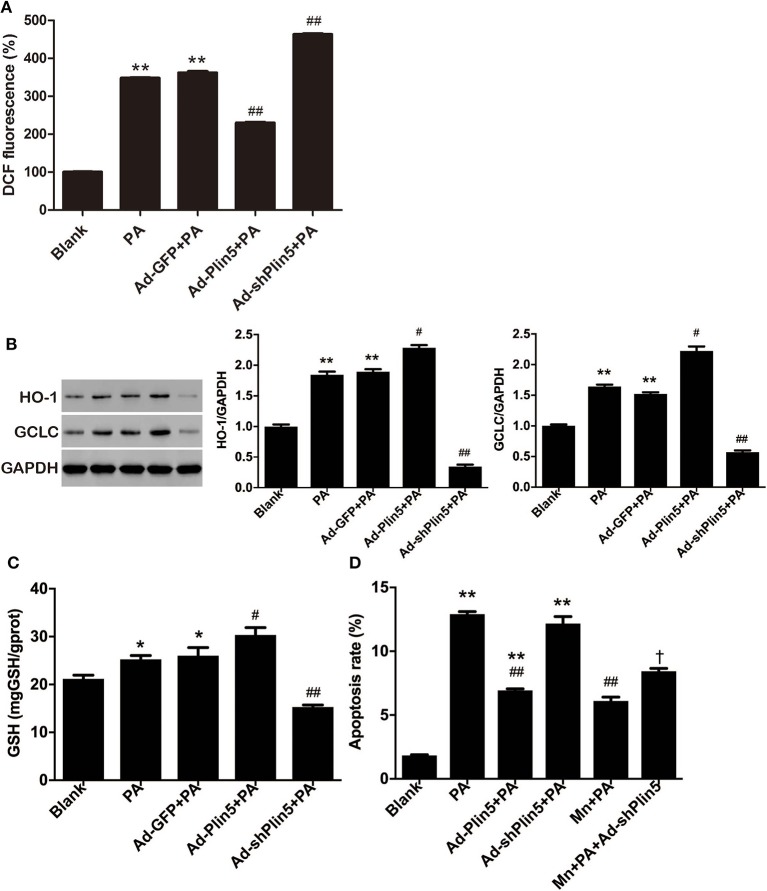
The potential role of Plin5 in the regulation of ROS production and oxidative stress defense in INS-1 β-cells with lipid overload. **(A)** Fold induction of DCF fluorescence in INS-1 cells with or without transfecting Ad-GFP (24 h), Ad-Plin5 (24 h), or Ad-shPlin5 (36 h) and exposed to 0.5 mM palmitate (PA) for 24 h. The value of untreated cells (Blank) was set to 100%, and the other values were presented as relative to that. Data are expressed as the mean ± SEM (*n* = 3); **P < 0.01 against Blank; ^*##*^*P* < 0.01 against PA group. **(B)** Western blot analysis of HO-1 and GCLC in INS-1 cells treated as described in **(A)**. Data are presented as the mean ± SEM (*n* = 3) and expressed as the fold change taking Blank group as 1. GAPDH was used as an invariant control for equal protein loading. **P < 0.01 against Blank; ^#^*P* < 0.05, ^*##*^*P* < 0.01 against PA group. **(C)** GSH content in INS-1 cells treated as described in **(A)**. Data are expressed as the mean ± SEM (*n* = 3); *P < 0.05 against Blank group; ^#^*P* < 0.05, ^*##*^*P* < 0.01 against PA group. **(D)** Apoptosis of INS-1 cells transfected with Ad-Plin5 or Ad-shPlin5 for indicated time and treated with palmitate (PA) and/or 25 μM of MnTMPyP (Mn) for 24 h. Data are expressed as mean ± SEM (*n* = 3); **P < 0.01 against Blank group; ^*##*^*P* < 0.01 against PA group; ^†^*P* < 0.05 against Ad-shPlin5+PA group. Mn: MnTMPyP.

There is some evidence that oxidative stress may activate intrinsic apoptosis pathways in pancreatic β-cells [reviewed by (35)]. As Plin5 has been shown to negatively associate with lipid overload-induced oxidative stress in several tissues of mice (21–23), we further investigated the potential induction of antioxidant cellular defenses by Plin5. To this end, INS-1 cells were incubated with palmitate, a physiological inducer of oxidative stress, and ROS production, protein expressions of antioxidant enzymes GCLC and HO-1 were detected. As shown in Figure 2A, DCF fluorescent signal, which represented a measure of generalized oxidant production, was increased by 3.5-fold after 24 h palmitate incubation; this induction was inhibited by *Plin5* overexpression and augmented by *Plin5* knockdown. The protein levels of GCLC and HO-1 were mildly upregulated by palmitate and further enhanced in *Plin5*-overexpressing cells (Figure 2B), but this augmentation was abolished in *Plin5*-depleted cells. As GCLC is an enzyme responsible for the synthesis of GSH (36), an important antioxidant, GSH content was then determined in INS-1 cells. Again, upregulation of *Plin5* significantly increased the cellular GSH level as compared with palmitate group, whereas deficiency of *Plin5* decreased GSH content, which was even lower than that of palmitate group (Figure 2C). Based on these results, we conclude that Plin5 has a notable antioxidative effect against oxidative stress, a pathway previously implicated in β-cell lipotoxicity.

### Anti-apoptotic Role of Plin5 Is Partially Mediated by Antioxidative Effect

Given that pancreatic β-cells are extremely sensitive to oxidative stress because of the relatively low antioxidant defenses (5), it has been suggested that FFA may induce β-cell apoptosis by increasing oxidative stress (37). To test whether altering *Plin5* mediates lipotoxic cell death via modulating oxidative stress, we used the O^2−^ scavenger MnTMPyP. Treatment with 25 μM MnTMPyP for 24 h partially protected INS-1 cells from palmitate and decreased the proportion of apoptotic cells to the level as observed in palmitate+Ad-Plin5 group. Moreover, MnTMPyP also partially rescued *Plin5* silencing-mediated apoptosis in palmitate treated INS-1 cells (Figure 2D). Thus, our data suggest that the attenuated oxidative stress contributes to Plin5 protective effect in the context of lipotoxicity.

### Plin5 Induces an Nrf2-ARE Dependent Antioxidative Stress Defense by PI3K/Akt and ERK Signaling Pathways

**Figure 3:**
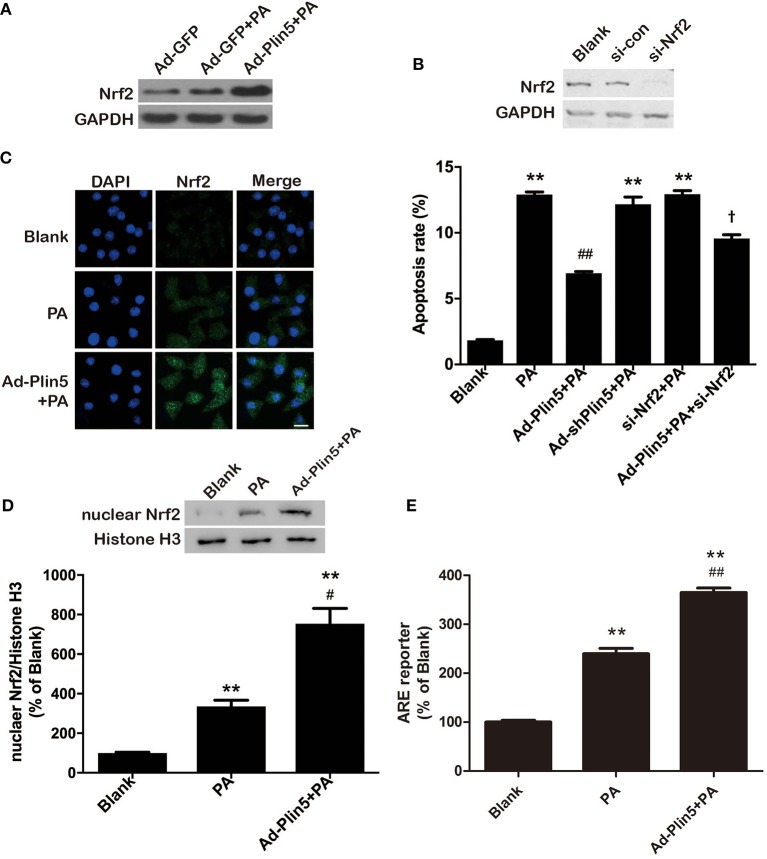
Plin5 activates Nrf2-ARE dependent antioxidative pathway in INS-1 cells. **(A)** Expression of Nrf2 in INS-1 cells treated with Ad-GFP or Ad-Plin5 and exposed to 0.5 mM palmitate (PA) for 24 h. Total cell lysates were analyzed by Western bolt analysis and GAPDH expression was used as an internal control. Representative blot for 3 independent experiments is presented. **(B)** Apoptosis of INS-1 cells transfected with Ad-Plin5, Ad-shPlin5, Nrf2 siRNA, or Ad-Plin5+Nrf2 siRNA and then treated with PA for 24 h (lower side). Nrf2 deletion by siRNA was confirmed in the upper side. Data of apoptosis rate are expressed as the mean ±SEM (*n* = 3); **P < 0.01 against Blank group; ^*##*^*P* < 0.01 against PA group; ^†^*P* < 0.05 against Ad-Plin5+PA group. **(C)** Analysis of nuclear translocation of Nrf2 in INS-1 cells transfected with Ad-Plin5 and exposed to PA for 24 h. After fixation and permeabilization, the cells were stained with specific antibodies against Nrf2 (green), followed by staining with DAPI (blue). Representative images from 3 independent experiments are presented. Scale bar = 20 μm. **(D)** Protein levels of nuclear Nrf2 in INS-1 cells transfected with Ad-Plin5 and exposed to PA for 24 h. Up: representative Western blot image. Down: densitometric results of the fold induction relative to Histone H3 expressed as percentage relative to untreated cells (Blank). Data are expressed as the mean ± SEM (*n* = 3). **P < 0.01 against Blank group; ^#^*P* < 0.05 against PA group. **(E)** ARE reporter activity in INS-1 cells. Cells were co-transfected with the ARE-luciferase report construct (750 ng) and the Renilla luciferase reporter vector plasmid pRL-TK (50 ng) using Lipofectamine 2000 for 6 h. After that, the cells were transfected with Ad-Plin5 for indicated time and then 0.5 mM palmitate was added for 24 h. The luciferase activity of cell lysates was measured using a GloMax®-Multi + Detection System and was expressed as relative luciferase/Renilla activity. Data are expressed as the mean ± SEM (*n* = 3); **P < 0.01 against Blank group; ^*##*^*P* < 0.01 against PA group. The value of Blank was set to 100%, and the other values were presented as relative to that.

Induction of oxidative stress in pancreatic β-cells leads to the activation of Nrf2 (38), previously shown to regulate antioxidant genes such as *Gclc* and *Ho-1* (36). Considering that Nrf2 is a master regulator in the cellular adaptive response to oxidative stress (38, 39), we then examined whether Nrf2 was involved in the antioxidant effect of Plin5. Palmitate treatment in INS-1 β-cells mildly induced Nrf2 expression, which was further enhanced by overexpression of *Plin5* (Figure 3A). *Plin5* overexpression *per se* had no effect on Nrf2 level (data not shown). Next, we asked if Nrf2 expression is required for Plin5-mediated rescue of β-cells. As shown in Figure 3B, Nrf2 depletion failed to restore the cytoprotection from lipotoxicity conferred by Plin5 in INS-1 cells. As Nrf2 target protein GCLC and HO-1 were modulated by Plin5, and Nrf2 controls the expression of cellular antioxidant and detoxifying enzymes by binding to ARE in their promoter regions (39). We presumed that Plin5 may truly affect transcriptional activity through Nrf2 activation and binding to ARE. A reporter gene assay was performed by transfecting with the pGL4.37[luc2P/ARE/Hygro] Vector. As expected, adenovirus-mediated *Plin5* overexpression resulted in significant Nrf2 translocation to nucleus in INS-1 cells exposed to palmitate (Figures 3C,D). While exposure of β-cells to palmitate enhanced ARE reporter activity by 2.4-fold, this effect was further enhanced in *Plin5*-overexpressing cells (Figure 3E). Hence, our data presented herein confirm that the Nrf2-ARE pathway was positively regulated by Plin5 in INS-1 β-cells.

**Figure 4:**
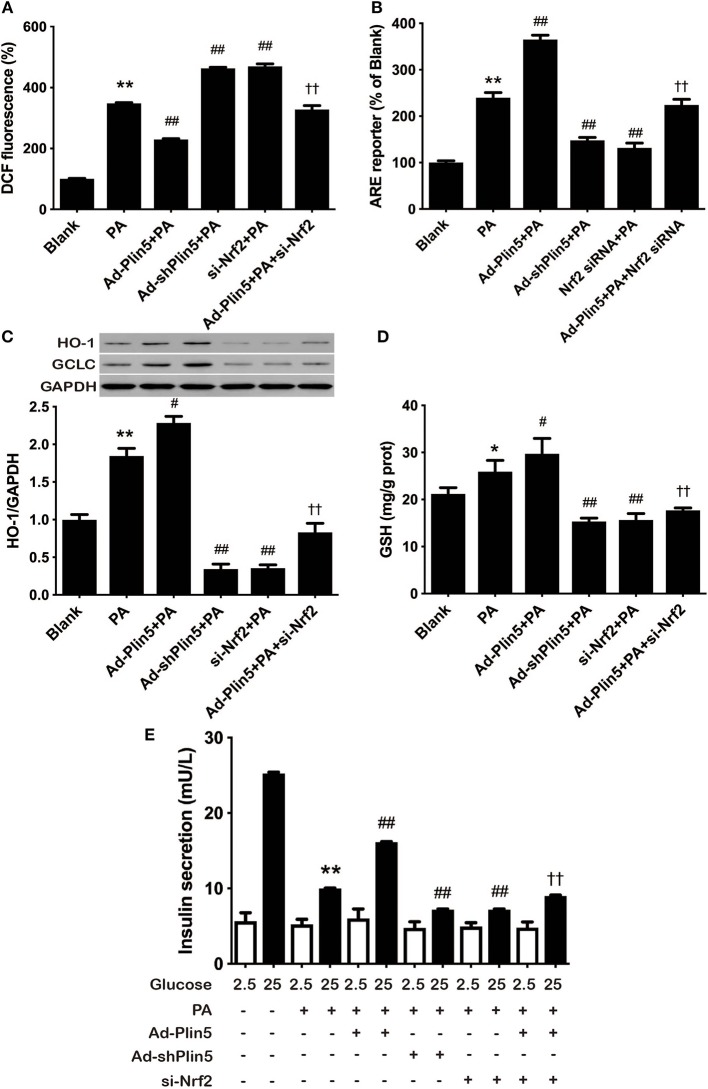
Nrf2 knockdown inhibited the antioxidative effect of Plin5. **(A)** Changes in DCF fluorescence in INS-1 cells. INS-1 cells transfected with Ad-Plin5, Ad-shPlin5, Nrf2 siRNA or Ad-Plin5+Nrf2 siRNA were incubated with 0.5 mM palmitate (PA) for 24 h and then DCF fluorescence was quantified using a flow cytometry. The value of PA group was set at 100%, and other values were presented as relative to that. Data are expressed as the mean ± SEM (*n* = 3); **P < 0.01 against Blank group; ^*##*^*P* < 0.01 against PA group; ^††^*P* < 0.01 against Ad-Plin5+PA group. **(B)** ARE reporter activity in INS-1 cells. INS-1 cells were co-transfected with the ARE-luciferase report construct (750 ng) and the Renilla luciferase reporter vector plasmid pRL-TK (50 ng) using Lipofectamine 2000 for 6 h. After that, the cells were transfected with different adenoviruses and (or) Nrf2-siRNA for 18 h and then 0.5 mM PA was added for 24 h. The luciferase activity of cell lysates was measured using a GloMax®-Multi + Detection System and are expressed as relative luciferase/Renilla activity. Data are expressed as the mean ± SEM (*n* = 3); **P < 0.01 against INS-1 cells exposed to Blank group; ^*##*^*P* < 0.01 against PA group; ^††^*P* < 0.01 against Ad-Plin5+PA group. **(C)** HO-1 and GCLC protein expression in INS-1 cells treated as described in **(A)**. Protein expressions were determined by Western blots. Data are presented as the mean ± SEM (*n* = 3) and expressed as the fold change taking PA group as 1. GAPDH was used as an invariant control for equal protein loading. **P < 0.01 against INS-1 cells exposed to Blank group; ^#^*P* < 0.05 against PA group; ^††^*P* < 0.01 against Ad-Plin5+PA group. **(D)** GSH content in INS-1 cells treated as described in **(A)**. Data are expressed as the mean ± SEM (*n* = 3); *P < 0.05 against INS-1 cells exposed to Blank; ^#^*P* < 0.05, ^*##*^*P* < 0.01 against PA group; ^††^*P* < 0.01 against Ad-Plin5+PA group. **(E)** Glucose stimulated insulin secretion in INS-1 cells treated as described in A. Data are expressed as the mean ± SEM (*n* = 3); **P < 0.01 against Blank INS-1 cells at 25 mM glucose; ^*##*^*P* < 0.01 against PA cells at 25 mM glucose; ^††^*P* < 0.01 against Ad-Plin5+PA group.

Finally, to determine whether the antioxidative effect of Plin5, closely associated with β-cell dysfunction and apoptosis, is mediated by Nrf2, we examined ROS production by *Nrf2* silencing. Nrf2 siRNA transfection abolished the protective effect of Plin5 on ROS production (Figure 4A). Supporting the pivotal role of Nrf2 in β-cell antioxidative stress defenses, silencing *Nrf2* inhibited the induction of ARE reporter activity (Figure 4B) and antioxidant enzymes expression (Figure 4C) by Plin5. In keeping with this, the elevation of GSH level by *Plin5* overexpression was abolished in *Nrf2*-deficient cells (Figure 4D). Moreover, in order to investigate the functional effects of *Nrf2* knockdown on the regulation of insulin secretion, GSIS was measured. INS-1 cells treated with Ad-Plin5 were protected from palmitate induced decrease of insulin secretion, but the knockdown of *Nrf2* blocked the protective effects (Figure 4E). Overall, these results suggest that Nrf2 activation by Plin5 induces a prosurvival antioxidative response, which contributes to the protective effects in β-cell dysfunction by oxidative stress.

**Figure 5:**
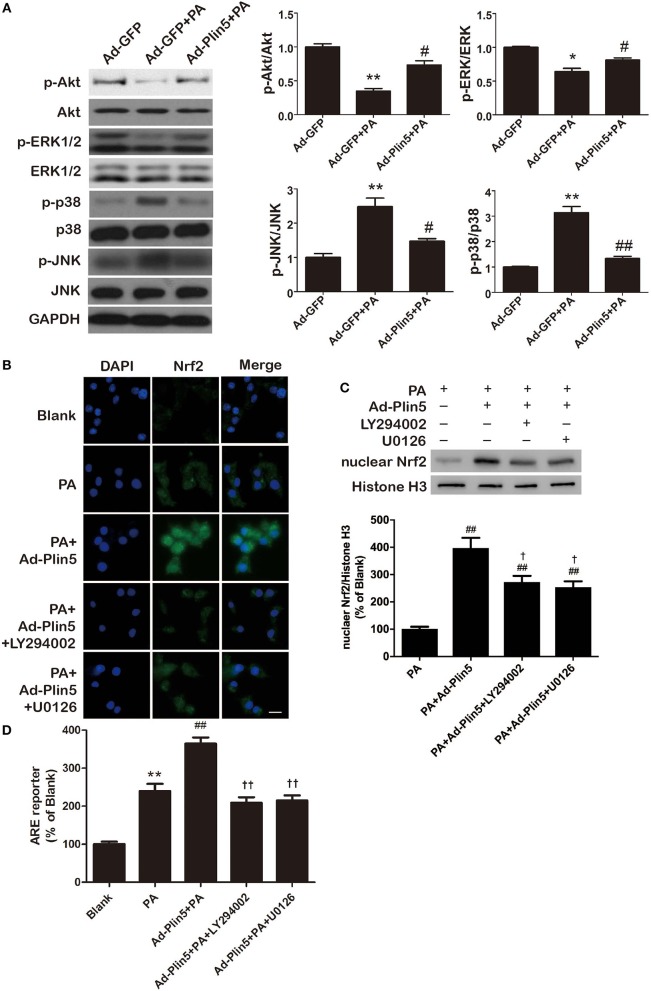
Plin5 antioxidant effect is PI3K/Akt and ERK dependent and involves Nrf2-ARE system. **(A)** The activity of PI3K/Akt and MAPKs pathway in INS-1 cells treated with Ad-GFP, Ad-GFP+palmitate (PA) or Ad-Plin5+PA for 24 h. The protein expressions of p-Akt, Akt, p-ERK1/2, ERK1/2, p-p38, p38, p-JNK, and JNK were detected by Western blots (left). Data are presented as the mean ± SEM (*n* = 4) and expressed as the fold change taking Ad-GFP group as 1 (right). GAPDH was used as an invariant control for equal protein loading. *P < 0.05, **P < 0.01 against Ad-GFP; ^#^*P* < 0.05, ^*##*^*P* < 0.01 against Ad-GFP+PA. **(B)** Nuclear translocation of Nrf2 in INS-1 cells. INS-1 cell transfected with or without Ad-Plin5 were pretreated for 1 h with either PI3K/Akt inhibitor LY294002 or ERK inhibitor U0126 and then incubated with palmitate for 24 h. After fixation and permeabilization, the cells were stained with specific antibodies against Nrf2 (green), followed by staining with DAPI (blue). Representative images from 3 independent experiments are presented. Scale bar = 20 μm. **(C)** Protein levels of nuclear Nrf2 in INS-1 cells treated as described in **(B)**. Up: representative Western blot image; Down: densitometric results of the fold induction relative to Histone-H3 expressed as percentage relative to PA group. Data are expressed as the mean ± SEM (*n* = 3). ^*##*^*P* < 0.01 against PA group; ^†^*P* < 0.05 against Ad-Plin5+PA group. **(D)** ARE reporter activity in INS-1 cells treated as in **(B)**. Data are expressed as the mean ± SEM (*n* = 3); **P < 0.01 against Blank group; ^*##*^*P* < 0.01 against PA; ^††^*P* < 0.01 against Ad-Plin5+PA group.

In the aortic tissues, heart and liver, Plin5 has been reported to associate with PI3K/Akt or MAPK pathway (p38 MAPK, ERK, and JNK) (21, 24, 40), which involve in modulating oxidative stress (41, 42). Here, we first investigated the protein expressions of p-Akt, p-p38, p-ERK1/2, and p-JNK in INS-1 cells incubated with palmitate. As shown in Figure 5A, we found that palmitate treatment decreased the expression of p-Akt and p-ERK1/2 but induced the level of p-p38 and p-JNK in INS-1 cells. Of note, Plin5 gain-of-function restored the expression of p-Akt and p-ERK1/2 and blocked the elevation of p-p38 and p-JNK, indicating the increased activity of PI3K/Akt and ERK pathways and decreased activity of p38 and JNK pathways (Figure 5A). Following, to determine whether the activated pathways are involved in Plin5-mediated Nrf2 activation in β-cells, INS-1 cell transfected with Ad-GFP or Ad-Plin5 were pretreated for 1 h with either LY294002 (PI3K/Akt inhibitor) or U0126 (ERK inhibitor) and then incubated with palmitate for 24 h. We found that either LY294002 or U0126 treatment could reduce Plin5-induced nuclear Nrf2 translocation (Figures 5B,C) and ARE reporter activity (Figure 5D). These data demonstrate that PI3K/Akt and ERK pathways may play a specific role in Plin5-induced antioxidant defense by activation of Nrf2-ARE system in INS-1 cells.

## Discussion

Oxidative stress has been proposed as a common mechanism suggested for disruption of pancreatic β-cell, a process which is critical in the pathophysiology of type 2 diabetes (43). Plin5, a LD protein which is abundantly expressed in tissues with high oxidative capacity, is able to improve the cellular antioxidant capacity of these tissues and in turn partly prevent associated injuries (22–24, 44). Recently, Plin5 has been established as a LD protein important for lipid metabolism and insulin secretion in β-cells (27). And our previous data showed that Plin5 alleviated nutrition overload-induced endoplasmic reticulum stress in pancreatic β-cells (26). However, whether Plin5 may also modulate oxidative stress and alleviate oxidative injuries in pancreatic β-cells, the hypersensitive targets of oxidative damage, and the detailed molecular mechanisms involved are not understood.

**Figure 6:**
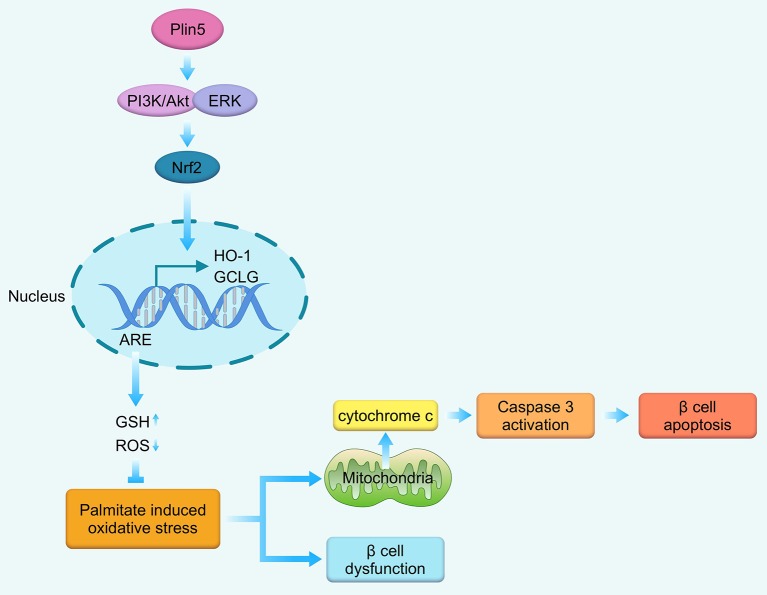
Schematic representation of the main findings of this study. In the context of palmitate-induced lipotoxicity, Plin5 activates PI3K/Akt and ERK signaling, which induces Nrf2 translocation to the nucleus where it binds with ARE, enhances the transcription of down-stream antioxidant enzymes GCLC and HO-1. Then, the antioxidant defense system is enhanced as manifested by decrease in cellular ROS production and elevated in GSH level. These cascade events ultimately contribute to the protective effects against mitochondrial-associated apoptosis and β-cell dysfunction. GCLC, glutamate-cysteine ligase catalytic subunit; GSH, glutathione; HO-1, heme oxygenase-1; ROS, reactive oxygen species.

We presently demonstrate that Plin5 is an important prosurvival factor in a pancreatic β-cell line INS-1 cells facing lipotoxic conditions. By inducing Nrf2 expression and triggering the Nrf2 translocation, consequently binding with ARE and upregulating antioxidative enzymes expression and GSH levels under lipotoxic stress, Plin5 equips β-cells with an effective antioxidative stress defense against the deleterious effects of palmitate including the activated mitochondrial pathway of apoptosis and impaired β-cell function. Moreover, the role of Plin5 in antioxidant defense of β-cells is PI3K/Akt and ERK pathways dependent as blocking these pathways by chemical inhibitors prevented Plin5-induced nuclear Nrf2 translocation and ARE reporter activity. In this *in vitro* study, we establish for the first time the significance of Plin5 with antioxidant capacity for preventing oversupply of palmitate-mediated lipotoxicity in pancreatic β-cells (Figure 6) and might be a potential therapeutic target for T2D.

While this is the first study to demonstrate the relationship between Plin5 and oxidative stress in pancreatic β-cells, it is not surprising as Plin5 has been reported to be an important factor in maintaining cellular metabolic homeostasis during various kinds of stress and negatively associated with oxidative stress in non-insulin-secreting cells (21, 23, 24, 44). Indeed, in HepG2 cells, overexpression of Plin5 protects against hydrogen peroxide or lipopolysaccharide induced cellular oxidative stress (44). In mouse hearts, Plin5 deficiency provokes oxidative stress, leads to exacerbated myocardial ischaemia/reperfusion injury (24). However, the exact mechanisms involved in the protective role of Plin5 are not fully understood. There is some limited evidence indicating that Plin5 may enhance the function of mitochondria, an organelle which is responsible for the majority of cellular ROS production (44). By gain-of-function strategy, Plin5 was observed to promote mitochondria-LD contact, up-regulated mitochondria function-related genes and hence enhancing mitochondrial function in HepG2 cells. The author concluded that the Plin5-mediated mitochondria-LD contact could be an important mechanism for cells to respond to oxidative stress (44). Similarly, Plin5 overexpression in brown adipose tissue also recruits mitochondria to LD and then increases ATP synthase-dependent triglyceride synthesis coupled with reduced fatty acid oxidation capacity (45). While, in the context of Plin5 deficiency, mitochondria was severely damaged with reduced mitochondrial oxidative capacity (46), and provoked mitochondrial proliferation possibly as a result of a compensatory regulatory mechanism of mitochondria dysfunction (47). A major finding of the current study is that Nrf2 acts as a key node downstream of Plin5 that links Plin5 to alterations in antioxidant defense. Our present report, for the first time, shed new light on the underlying mechanisms of Plin5 in alleviating oxidative stress. As we know, Nrf2 which is activated by oxidative stress acts as a critically important regulator of antioxidant response by modulating expression of ARE-containing genes, including enzymes involved in hydrogen peroxide scavengers, GSH biosynthesis and chemical detoxification (39). Recently, Yagishita and colleagues presented detailed *in vivo* evidence to demonstrate that Nrf2 induction prevents reactive species damage including oxidative DNA-adduct formation, impaired insulin secretion, and apoptosis in pancreatic β-cells (38). Furthermore, activation of the Nrf2-antioxidant pathway is reported to be a possible mechanism mediating self-repair of β-cells after a short-term exposure to a high fat diet feeding (48). Our present findings identify Plin5 as a positive regulator of Nrf2 in β-cells under lipid stress. We observed that Plin5 induced the expression of Nrf2, enhanced its nuclear import and prosurvival Nrf2-ARE signaling in β-cells. Overexpression of *Plin5* increased and knock-out of *Plin5* decreased expression of Nrf2-targeted enzymes and level of antioxidant molecular GSH under lipotoxic conditions. And the anti-oxidative effect was Nrf2-dependent because silencing *Nrf2* by si-Nrf2 weakened the modulation of Plin5 in antioxidant defense and the protective action on INS-1 β-cells. Whether Plin5 directly interacts with Nrf2 remains to be examined since a previous report described Plin5 as a nuclear protein involved in mediating oxidative function (32). Furthermore, studies aimed at identifying the effect of Plin5 on Nrf2 degradation are ongoing in light of the very short half-life of Nrf2 protein.

The detailed mechanism of the antioxidant role of Plin5 via regulating Nrf2-ARE system in β-cells was explored preliminary in the present study. In some tissues, Plin5 has been reported to involve in the regulation of PI3K/Akt or MAPK pathways (p38 MAPK, ERK, and JNK) (21, 24, 40, 44, 49), which is the upstream modulators of Nrf2 in pancreatic β-cells (39, 41). As evidenced by prior studies, excess ROS production and oxidative stress results in the activation of apoptotic p38 MAPK and JNK signaling, whereas activation of antiapoptotic PI3K/Akt and ERK pathways facilitates the translocation of Nrf2 into the nucleus, thereby activating the Nrf2/ARE pathway and alleviating oxidative damage (50, 51). In the current study, p38 MAPK and JNK pathways were found to be activated but PI3K/Akt and ERK pathways were inhibited in β-cells under palmitate induced-oxidative stress. However, *Plin5* overexpression dramatically alleviated oxidative stress and prevented the disturbances in PI3K/Akt and MAPK pathways which were provoked by nutrition overload. Moreover, we found that the antioxidant role of Plin5 was PI3K/Akt and ERK signaling pathways dependent as Plin5-induced activation of Nrf2-ARE system was significantly inhibited by pharmacologic inhibitor of PI3K/Akt or ERK. In line with our data in pancreatic β-cells, livers of mice with liver-specific *Plin5* ablation exhibited activation of JNK and *Plin5*-null cardiomyocytes showed decreased phosphorylation of PI3K/Akt (24, 40). However, in the aortic tissues, *ApoE/Plin5* double knockout linked with the activation of PI3K/Akt and MAPK pathways, therefore, may contribute to oxidative stress generation (21). The reason of discordant results is not well known, the induction of PI3K/Akt and MAPK pathways by *ApoE* deletion *per se* may complicate the study of the regulation of Plin5 in these signaling pathways and tissue-specific role of Plin5 in different target tissues may also be considered. More recently, Tan et al. identified that the ROS-JNK-p38-ATFs regulatory axis up-regulated the expression of Plin5 so that it alleviated the cellular oxidative stress induced by hydrogen peroxide or lipopolysaccharide through reducing the generation of ROS products by mitochondria in HepG2 cells. Considering the positive regulation of Plin5 on p38 MAPK and JNK activities revealed in the present data, we speculated that elevation of ROS-JNK-p38-ATFs pathway on Plin5 may serve as a negative feedback to inhibit the activation of JNK and p38 and ultimately alleviate oxidative damage, although further investigation is needed. Overall, the results above indicate that Plin5 exhibits protective effects against oxidative stress via Nrf2-ARE pathway in INS-1 β-cells by activating PI3K/Akt and ERK pathways. However, one key limitation of our study is that all the data were obtained through *in vitro* experiments, which needs to be further verified *in vivo* in the future.

In pancreatic β-cells, lipotoxicity can impact cell survival, as well as β-cell function, manifested by reduced GSIS (3, 4). Intriguingly, in the present study we revealed that Plin5 can rescue impaired GSIS under the condition of lipid stress via enhanced antioxidant machinery. The relation of GSIS and ROS is intricate. One hand, ROS, in particular hydrogen peroxide, may function as physiological signaling molecules mediating a variety of physiological processes such as GSIS in β-cells (52). However, elevated ROS can also activate some stress-sensitive pathways that have been linked to decreased insulin secretion (37). Among these pathways, activation of the NF-κB and JNK/STAT pathways has been associated with ROS-mediated pancreatic β-cell death and therefore decreased GSIS (53). Whether these pathways are also involved in Plin5-mediated antioxidant effect in lipid overloaded pancreatic β-cells remains to be examined, whereas Plin5 indeed modulates NF-κB pathway in the brachiocephalic arteries of ApoE^−/−^ mice (21). The other hand, excessive and persistent production of ROS directly oxidizes and damages DNA, proteins, and lipids and causes β-cell dysfunction and apoptosis (37). In addition, insulin release required increased ATP production during glucose metabolism in pancreatic β-cells (54). Continuous ROS production (endogenous or exogenous) as occurring in the oxidative stress condition can lead to ATP depletion, therefore limiting GSIS as well as causing β-cell dysfunction (55). At last, similar to other cell types, β-cells also use FFA lipolysed from adipose tissue as the principal energy source to maintain cell functions such as GSIS and basal lipolysis is critical for normal GSIS (56). Higher expression of Plin5 in β-cells favors lipolysis and fatty acid oxidation in the presence of FFA (27), although the associations of Plin5 and lipid metabolism in non-insulin-secreting cells are controversial, likely due to unique interaction of Plin5 with multiple lipolytic regulators (24, 57). Thus, Trevino et al. demonstrated that overexpression of Plin5 in islets enhanced the augmentation of GSIS by acute FFA treatment (1 h) via regulating intracellular lipid metabolism (27). Collectively, the possible activities of Plin5 as regulators of some pathways, antioxidant defenses and lipid metabolism in β-cells may ensure a more efficient removal of oxidant species with no negative impact on GSIS.

Interestingly, we found that 24 h palmitate treatment also induced a cellular adaptive antioxidant response in INS-1 β-cells, as evidenced by mild activation of Nrf2-ARE pathway and elevation of GSH level (Figures 3–4), although this response couldn't completely overcome the challenges of increased oxidative stress. Our results are consistent with previous studies conducted in not only β-cells (58) but also in other type of cells (59). This phenomenon may be explained by our previous observation that Plin5 expression is also moderately induced by palmitate treatment (26) and hence elevated Plin5 expression may contribute to the antioxidant response in β-cells, although the details are still inexplicit. While, this adaptive response which may represent an attempt to minimize palmitate's prooxidant actions is not sufficient to overcome palmitate-mediated oxidative damage, which ultimately leads to a dramatic elevation of intracellular ROS levels. However, overexpression of Plin5 induced significant antioxidant defense and alleviated oxidative damage induced by palmitate overload.

In conclusion, our results demonstrate a critical role for Plin5 in induction of antioxidant defense and alleviation of oxidative damage in INS-1 pancreatic β-cells. Additionally, we highlight a novel mechanism of Plin5 in regulating oxidative stress which involves PI3K/Akt and ERK dependent activation of Nrf2-antioxidant pathway.

## Data Availability Statement

The raw data supporting the conclusions of this article will be made available by the authors, without undue reservation, to any qualified researcher.

## Author Contributions

YZhu contributed with funding acquisition, project administration, experiments conduction, data analysis and interpretation, manuscript writing and revision, final approval of manuscript and guarantor of this work. CR contributed with experiments conduction, data analysis. MZ contributed with data analysis and figure preparation. YZho contributed with project administration and manuscript revision.

### Conflict of Interest

The authors declare that the research was conducted in the absence of any commercial or financial relationships that could be construed as a potential conflict of interest.
